# Development and Psychometric Properties of a Questionnaire Assessing Self-Reported Generic Health Literacy in Adolescence

**DOI:** 10.3390/ijerph17082860

**Published:** 2020-04-21

**Authors:** Olga Maria Domanska, Torsten Michael Bollweg, Anne-Kathrin Loer, Christine Holmberg, Liane Schenk, Susanne Jordan

**Affiliations:** 1 Department of Epidemiology and Health Monitoring, Robert Koch Institute, 12101 Berlin, Germany; LoerA@rki.de (A.-K.L.); JordanS@rki.de (S.J.); 2 Centre for Prevention and Intervention in Childhood and Adolescence (CPI), Faculty of Educational Science, Bielefeld University, 33615 Bielefeld, Germany; torsten.bollweg@uni-bielefeld.de; 3 Institute of Social Medicine and Epidemiology, Brandenburg Medical School Theodor Fontane, 14770 Brandenburg an der Havel, Germany; christine.holmberg@mhb-fontane.de; 4 Institute of Medical Sociology and Rehabilitation Science, Charité – Universitätsmedizin Berlin, 10117 Berlin, Germany; liane.schenk@charite.de

**Keywords:** adolescents, health literacy, questionnaire, self-assessment, subjective measurement, validation, MOHLAA-Questionnaire

## Abstract

Health literacy is a promising approach to promoting health and preventing disease among children and adolescents. Promoting health literacy in early stages of life could contribute to reducing health inequalities. However, it is difficult to identify concrete needs for action as there are few age-adjusted measures to assess generic health literacy in young people. Our aim was to develop a multidimensional measure of health literacy in German to assess generic health literacy among 14- to 17-year-old adolescents, namely, the “Measurement of Health Literacy Among Adolescents Questionnaire” (MOHLAA-Q). The development process included two stages. Stage 1 comprised the development and validation using a literature review, two rounds of cognitive interviews, two focus groups and two rounds of expert assessments by health literacy experts. Stage 2 included a standard pretest (*n* = 625) of the questionnaire draft to examine the psychometric properties, reliability and different validity aspects. The MOHLAA-Q consists of 29 items in four scales: (A) “Dealing with health-related information (HLS-EU-Q12-adolescents-DE)”; (B) “Communication and interaction skills”, (C) “Attitudes toward one’s own health and health information”, and (D) “Health-related knowledge”. The confirmatory factor analysis indicated a multidimensional structure of the MOHLAA-Q. The internal consistency coefficients (Cronbach’s α) of the scales varied from 0.54 to 0.77. The development of the MOHLAA-Q constitutes a significant step towards the comprehensive measurement of adolescents’ health literacy. However, further research is necessary to re-examine its structural validity and to improve the internal consistency of two scales.

## 1. Introduction

Health literacy has been explored in numerous studies in adult populations. These studies have found that health literacy is associated with different health outcomes (e.g., health status, use of health services, mortality) [1] but is also an independent predictor of health status in addition to common sociodemographic factors such as age, income or education [2,3,4]. According to its broad, widespread definition, health literacy comprises the competencies, knowledge, and motivation to access, understand, appraise, and apply health-related information in order to make judgments and health decisions in everyday life [5]. The World Health Organization regards health literacy as “a critical determinant of health” as well as a resource that “must be an integral part of the skills and competencies developed over a lifetime, first and foremost through the school curriculum” [6].

In recent times, the promotion of health literacy as a strategy to reduce health disparities has been discussed, as limited health literacy is supposed to be an important predicator of health disparities [7]. Accordingly, promoting those skills in early stages of life should contribute to reducing health inequalities caused, among others, by a low health literacy level in the population. However, the evidence on the exact nature of the relationship between health literacy and health disparities remains still scarce [7]. This is because there is a lack of studies that examine the dynamic, contextual nature of health literacy and specify its level and change in the life span: in childhood, in adolescence and different phases of adult life.

Adolescence is an important life phase in terms of emotional, social, and cognitive developmental processes [8,9]. This phase of life is characterized by improvements in cognitive skills, information processing and cognitive self-regulation [10], which are basic skills for decision-making. Adolescents must manage different developmental tasks, such as developing their autonomy, their own norms, and values [8] as well as assuming responsibility for their lives and health. At the same time, adolescents assess their vulnerability differently than adults do [11,12] by underestimating risk factors and consequences of their health-related actions. Thus, adolescents are prone to risky behavior (e.g., unprotected sexual activity, dangerous driving, and substance use). In addition, adolescents are growing up in the modern information and digital society with easy and ubiquitous access to health information, health apps and devices, and they often become the target group for health-related services [13]. Health literacy may therefore be particularly important in adolescence, as it involves the development of skills that go beyond theoretical and practical knowledge and involve the critical thinking, self-awareness and the skills required to be active citizens who take responsible actions to promote their own and others’ health [14].

Compared to the knowledge on adult populations, there is little knowledge about the level of health literacy and its distribution in children and adolescents and the importance of health literacy for health care, disease prevention, and health promotion in these population groups. Most previous studies and surveys in this population have been conducted in clinical or medical contexts or have focused on functional literacy or media literacy [15,16]. The lack of health literacy data for children and adolescents can be attributed to different causes, one of which is that a clear and commonly accepted definition and conceptual framework of health literacy in these age groups is lacking [17]. In addition, there is a lack of high-quality, valid, and age-adjusted measurements [18,19,20], followed by a lack of studies with children and adolescents [21].

In Germany, a few studies on adolescents’ health literacy have focused on the measurement of specific health literacy domains, such as health-related knowledge [22] or critical health literacy [23,24], or have targeted certain age groups of adolescents, e.g., 9- to 13-year-olds [25], 15- to 29-year-olds [26], or specific subgroups, such as educationally alienated young people [27] or adolescent and young adult cancer patients [28]. On the one hand, these studies provided insightful findings relating to those age groups and initiated the health literacy research among children and adolescents in this country. On the other hand, a research gap opened up in Germany regarding a measurement tool assessing generic health literacy of young people in mid-adolescence. To close this gap, the “Measurement of Health Literacy Among Adolescents (MOHLAA)” project aimed to develop and validate an age-adjusted instrument in the German language to assess self-reported generic health literacy. The project was conducted as part of the German Health Literacy in Childhood and Adolescence (HLCA) Consortium [29].

We focused on young people in mid-adolescence (adolescents aged 14–17 years) as they start to make their own health-related decisions, while patterns of increased risky behavior can also be observed [30,31]. Our aim was to develop a self-administered, paper-and-pencil measurement tool applicable for the monitoring and evaluation of health literacy in population-based studies. This paper describes the development process of the MOHLAA-Q, with a focus on evaluating the instrument’s applicability for the target group and its psychometric properties.

## 2. Materials and Methods

**Figure 1:**
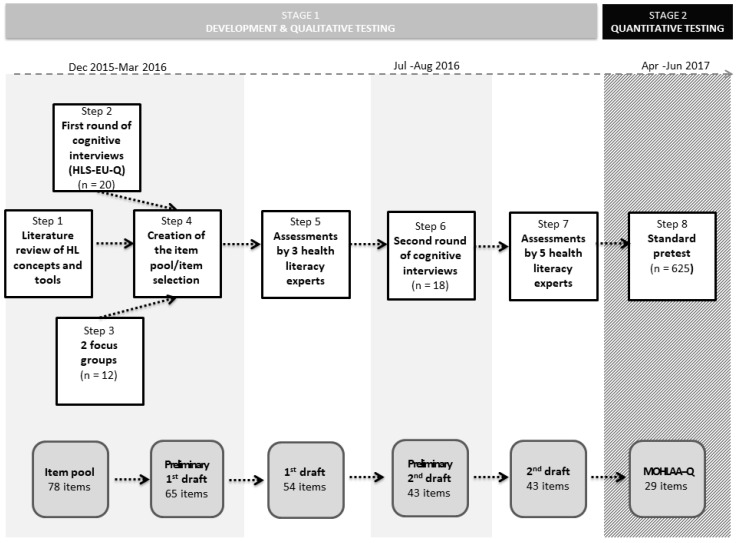
Phases of the development process of the MOHLAA-Q applied in the MOHLAA study (2015–2018) in Germany.

The development process of the MOHLAA-Q, as shown Figure 1, consisted of two stages: the development and qualitative testing of the 1st and 2nd drafts of the MOHLAA-Q (stage 1) and quantitative testing of the 2nd draft with a standard pretest and finalization of the MOHLAA-Q (stage 2).

### 2.1. Stage 1: Development and Qualitative Testing

Stage 1 comprised a literature review (LR) (step 1), two rounds of cognitive interviews (CIs) (step 2 and 6) and focus groups (FGs) (step 3), both with adolescents aged 14–17, the creation of the item pool (step 4) and two rounds of expert assessments (EAs) by health literacy experts (step 5 and 7). Here, we only describe the most important steps, a detailed description of the methods and results of the first-round CIs and the FGs can be found elsewhere [32,33].

At step 1, we conducted an LR on definitions, models and concepts of health literacy in relation to adolescents to determine a conceptual framework for our questionnaire. The framework underlying the MOHLAA-Q is grounded on findings of a systematic review on definitions and models by Bröder et al. [17], in which health literacy of children and adolescents is regarded as “comprising variable sets of key dimensions, each appearing as a cluster of related abilities, skills, commitments, and knowledge that enable a person to approach health information competently and effectively and to derive at health-promoting decisions and actions” [17]. This multidimensional construct interrelates with “social and contextual determinants”. The key health literacy dimensions can to be clustered according to three core categories of individual attributes: (1) cognitive, (2) behavioral or operational, and (3) affective and conative. For example, all mental abilities and actions that enable a person to think, learn and process information are attributed to the cognitive category. In our final framework that we specified according to the findings of CIs and FGs (c.f. Section 3.1), we determined four theoretical dimensions (denoted as factors) of individual health literacy: cognitive (f1), behavioral (f2), behavioral/communicative (f3) and affective/conative competencies (f4). Furthermore, Bröder et al. highlight that families, peers and schools are major socialization agents in young people’s lives that influence their opportunities for being or becoming health literate. This was considered in the preliminary framework as a contextual factor (f5) of individual health literacy.

At this study phase, we also examined the literature on instruments to measure self-reported generic health literacy that had been developed exclusively for adolescents (13 to 18 years of age), or had been applied in these age groups. We determined the 47-item version of the European Health Literacy Survey Questionnaire (HLS-EU-Q47-GER [34]) as a blueprint. Its validity and reliability have been confirmed in many studies with adults [35,36,37,38]. Based on this instrument, we added scales and topics that more specifically considered adolescents’ health-related needs and characteristics [39]. To test the applicability of the HLS-EU-GER for measuring the generic health literacy of adolescents, CIs were applied (step 2). Simultaneously, we conducted two FGs (step 3) to explore in depth adolescents’ experiences of managing diseases, navigating health care services and promoting their own health. In step 4, the item pool and the 1st draft of the MOHLAA-Q was created. In the item pool, we included the age-group-relevant HLS-EU-Q47-GER items. We adapted items when feasible and added items from other instruments available in English or German that operationalized affective/conative and behavioral/communicative (i.e., communication and interaction skills) health literacy components, and health-related knowledge. Then, two project team members selected items from the item pool based on two criteria: appropriateness of the item language (plain and concise wording) and relevance of item content for the age group. To ensure the content validity of our tool, the preliminary version of the questionnaire was assessed by three researchers (experts) from the HLCA consortium (step 5) with different educational and professional backgrounds, including one teacher. They provided a qualitative assessment of the item language and relevance for operationalizing adolescents’ health literacy. The expert assessments resulted in the final 1st MOHLAA-Q draft. The main results of the development process were also discussed with the HLCA Scientific Advisory Board, which includes international health literacy experts. To test the 1st draft of the MOHLAA-Q, we once again conducted CIs (step 6) following the same procedure as in the 1st round of CIs [32] apart from the sampling and recruitment procedure. Subsequently, in step 7, five experts from the HLCA consortium scored the clarity of the wording of each item of the preliminary 2nd draft and the item relevance regarding the generic health literacy construct. The adaptation process resulted in the 2nd draft of the MOHLAA-Q. Methodological details of the mentioned developmental processes of stage 1 are described in Supplementary Materials Table S1.

### 2.2. Stage 2: Quantitative Testing

To test the feasibility and selected aspects of the criterion validity, construct validity and reliability (internal consistency) of the 2nd draft of the MOHLAA-Q, we conducted a standard pretest as step 8 [40].

#### 2.2.1. Pretest Design and Data Collection

In step 8, the pretest was conducted as a cross-sectional postal survey in the city of Berlin (Germany). A stratified random sample of addresses from four selected districts (Marzahn-Hellersdorf, Mitte, Neukölln und Friedrichshain-Kreuzberg) from the Berlin city resident register was used. These districts are characterized by a high proportion of people with immigration background and a heterogeneous social-economic structure of inhabitants. In our sample, we aimed to balance the distribution among four age groups (14- to 17-year-olds) and among girls and boys. The study participants received an incentive (voucher) with a value of 10 euros. The paper-and-pencil survey included 43 items from the 2nd draft of the MOHLAA-Q; other health literacy measures; a self-efficacy scale; questions regarding health behavior, subjective health status and use of health services; and some sociodemographic questions (details described below).

The study was approved by the Federal Commissioner for Data Protection and Freedom of Information, Germany. Participants, as well as their parents or legal guardians, were informed about the study objectives and the applicable data protection guidelines (anonymous data processing and record keeping). Written informed consent was obtained from each participant and her/his parents or legal guardians.

#### 2.2.2. Measures

##### 2nd Draft of the MOHLAA-Q

The 2nd draft of the MOHLAA-Q contained five scales. Table 1 shows topics of the scales (col. 1), a corresponding theoretical dimension (col. 2) and specified health literacy attributes (col. 3) which are operationalized in the respective scale. For example, scale A operationalizes two health literacy dimensions: a *cognitive* dimension that corresponds to understanding and appraising skills and a *behavioral* dimension that corresponds to information seeking and applying health-related information. The items of scales A–C were intended to measure subjective generic health literacy at the individual level. Scale D was intended to measure selected aspects of health-related knowledge. Scale E aimed at assessing respondents’ perceptions of the competencies of social agents (doctors, parents, friends, and persons in school settings) for communication and interaction about health-related issues. In our conceptual framework, scale E was assumed to represent a factor that substantially affected individual-related health literacy in adolescents. Response options in the scales A, B and E were designed as 4-point Likert-scales, in scale C as a 5-point Likert-scale and, in scale D as single choice with 5 response options (dichotomy coded). The higher values scored on the respective scales implied better individual-related subjective health literacy.

##### Further Scales for Validation

To examine the criterion (concurrent) and construct (convergent) validity of our instrument, we included in the survey established scales that measure subjective generic health literacy, functional health literacy, self-efficacy, social support, and one question about self-reported health status (scored as 1—very good, 2—good, 3—moderate, 4—bad, and 5—very bad). The first scale was the German version of the Health Literacy Assessment Tool (HLAT-8) [41], which assesses subjective generic health literacy in three health literacy domains (functional, interactive and critical) in the context of family and friends. In a Swiss study, the tool showed moderate internal consistency (Cronbach’s α = 0.64) and expected known-group validity (positive associations with one’s own and parental education). The tool consists of 8 items with 4- or 5-point Likert response scales and a differently formulated “does not apply” option (scored as 0). The valid responses are summed for a total HLAT-8 score with a range of 0–37 points.

The second scale was the Newest Vital Sign (NVS) test [42], which assesses basic reading and numeracy skills (functional heath literacy). The NVS showed high internal consistency (Cronbach’s α > 0.74), and its criterion validity was confirmed in many studies [43]. This frequently used tool has also been validated for adolescents [44,45]. The NVS was originally designed to be administered verbally; however, for the purpose of our study, the instrument was adapted as a self-administered, paper-and-pencil questionnaire. We used the German version of the NVS applied in the European Health Literacy Survey [34].

The third scale was a self-efficacy scale that measures the general construct of self-efficacy which was validated in many countries and for adolescents [46]. In the validation study with adolescents, Cronbach’s α > 0.78 was found and confirmatory factor analysis confirmed the one-factor structure of the scale [46]. The scale consists of 10 items that measure one’s confidence in the ability to master difficult situations, whereby success is attributed to one’s own competence.

The last scale was the Multidimensional Scale of Perceived Social Support (MSPSS) [47] as an indicator of social support. We applied two subscales, namely “Family” (4 items) and “Friends” (4 items) which showed good internal consistency in the validation study with adolescents (Cronbach’s α of 0.81 and 0.92, respectively [47]).

##### Sociodemographic Questions

The participants were categorized into four age groups (14-, 15-, 16-, and 17-year-olds). To assess their educational backgrounds according to the Berlin school system, we asked each participant whether he/she was attending school and which type of secondary school leaving certificate he/she planned to achieve (1—“I do not know yet”; 2—“Vocational Training Maturity (BBR)”; 3—“Advanced Vocational Training Maturity (eBBB)”; 4—“Intermediate Secondary School Leaving Certificate (MSA)”; 5—“Matriculation for a university of applied sciences”; 6—“High School Certificate (Abitur)”; 7—“Other school certificate, namely, (open ended)”). If the participant was not currently attending school, we asked which type of certificate she/he has already achieved (1—“Left school without a degree”; 2—“Certificate of secondary education (Hauptschulabschluss), Vocational Training Maturity (BBR)”; 3—“Advanced Vocational Training maturity (eBBR), Intermediate Secondary School Leaving Certificate (MSA), Secondary School Certificate (Realschulabschluss)”; 4—“Matriculation for a university of applied sciences, High School Certificate”; 5—“Other school certificate, e.g., acquired abroad”). We aggregated those data into one variable with three categories: (1) no high school certificate, (2) high school certificate, and (3) I do not know yet which degree I would like to achieve. Migration background (yes/no) was assessed with questions about the country of origin of both the participants and their parents. A participant who had at least one parent who was not born in Germany was regarded as a person with a migration background. To determine adolescents’ socioeconomic status, we used the six-item version of the Family Affluence Scale III (FAS) [48]. The responses to the items were given as specific values and calculated as an aggregated FAS index ranging from 0 to 13. The FAS index was divided into quintiles and categorized as follows: (1) low (1st quintile, <20% of the sample), (2) medium (2nd–4th quintiles, 20–80% of the sample) and (3) high (5th quintile, >80% of the sample) [49]. As a proxy indicator of parents’ education, we asked about the number of books in the adolescent’s home [50].

#### 2.2.3. Psychometric Analyses

First, we conducted item and reliability analyses. The aim of the item analysis was to identify the items and scales in the 2nd draft of the MOHLAA-Q that had poor psychometric properties, which would be excluded from the further analysis. For each item, we examined the missing values, distribution (mean, variance, skewness, and kurtosis) and item discrimination based on the corrected item-total (ITC) correlation [51]. No firm cutoffs for item variance have been suggested; however, one recommendation is to select items with higher variance [52]. We considered floor or ceiling effects to be present if >15% of the participants scored the lowest or highest possible score on the scales, respectively [53]. We also computed a difficulty index (DI) for each of the knowledge questions, which was calculated for each item as the percentage of correct answers divided by the number of responses. A high DI indicates an easy item, and a low percentage indicates a difficult item. In general, items should have DIs of no less than 20% and no greater than 80% [52].

In summary, in the first step of the analysis, we determined the following criteria for poorly performing items: a proportion of missing values over 5%, distribution characterized by substantial departure from normality (e.g., skewness = 2 and kurtosis = 7) [54], item discrimination values < 0.26 or DIs over 80% for the knowledge items.

To estimate the reliability of the scales, we examined internal consistency with Cronbach’s α coefficient for the multipoint scales (A, B, C and E) and the Kuder-Richardson (KR20) coefficient of reliability for dichotomous items (0-false, 1-true) in scale D. However, for scale D, we used a formative measurement model [55]. In this model, so-called cause indicators (items) can be independent from each other, as only the (latent) construct causally depends on each indicator. Therefore, reliability in the sense of internal consistency is not meaningful when indices are formed as a linear sum of measurements [56]. Accordingly, we assumed a low KR20 for scale D.

Second, we conducted confirmatory factor analysis (CFA) with the remaining items to examine the construct validity, more specifically, the structural validity of the single scales and the overall instrument consisting of scales A, B, and C. Scale D was specified in our conceptual framework as a single health literacy component operationalized as a formative measurement model [57]. In line with the finding of Schmidt et al. [25] and theoretical considerations about disentangling health knowledge from health literacy [58], we hypothesized a weak association of scale D with the other health literacy components. With the CFA, we examined the extent to which the empirical data fit the factor structure of the conceptual framework underlying the MOHLAA-Q. Additionally, we conducted CFA of scale A with the adapted HLS-EU-Q47-GER items with the same factor structures as those used in the HLS-EU-Q47-GER: a three-factor structure (perceived difficulty of dealing with health information in the contexts of (1) health care, (2) health promotion, and (3) disease prevention) and a four-factor structure (perceived difficulty of: (a) accessing, (b) understanding, (c) appraising, and (d) applying health information).

We used the robust weighted least squares mean-adjusted (WLSM) estimator for categorical data. The 1- or 2-factor model for each scale (cf. Table 1) was identified by fixing the variance of the latent variables. Finally, we examined the model fit for the three scales in relation to the four factors (cognitive, behavioral, behavioral/communicative, and affective/conative components) and for the overall model, where we assumed multidimensionality of the construct. The single scales were minorly revised to improve the goodness of fit and interpretability of the CFA model based on substantive justifications related to our specified model or poorly performing items (factor loading < 0.30). Modification indices were used to identify pairs of items within the scale that, if the error estimates were allowed to correlate, would improve the model fit and for which there appeared to be theoretically justifiable shared “method effects” [59]. We considered factor loadings and substantial changes related to the modification indices. To evaluate the model fit of the scales and the overall instrument, we considered the results of the chi-square test of model fit, χ^2^(df), with the *p*-value and the following fit indices, including the strict cutoffs for an good fit in parentheses [60]: Comparative Fit Index (CFI ≥ 0.96), Tucker-Lewis Index (TLI ≥ 0.95) [59,60], Root Mean Square Error of Approximation (RMSEA < 0.06) [61] and Weighted Root Mean square Residual (WRMR < 1.00) [62]. It has to be noted, however, that the goodness-of-fit indices are often affected by various aspects such as model complexity, estimation method, normality of data, and most notably, sample size (e.g., χ^2^ is inflated by sample size, and large N solutions are routinely rejected on the basis of χ2) and that they are only one aspect of model evaluation [59,62,63].

Finally, to assess the convergent validity, we examined the relationship of the final versions of scales A–C of the MOHLAA-Q with the other measures of health literacy (the HLAT-8 and NVS) and the self-efficacy scale. We expected a positive moderate correlation of scales A–C with the HLAT-8. As the NVS captures only certain basic attributes of generic health literacy (reading and numeracy skills), we expected a weak correlation of scale A with the NVS as shown in other studies [64,65], and no correlation with scale B and C. The self-efficacy scale was used specifically for the validation of scale C, which is intended to measure, among other factors, affective/conative health literacy components, such as health-related self-efficacy and motivation. There, we expected a weak to moderate positive association between the self-efficacy scale and scale C. To verify the concurrent validity, we examined the extent to which scales A–C were correlated with subjective health status. We expected a negative statistical association, as shown in studies with adults [66]. To validate scale D, we examined its relationship with the NVS and the question about the number of books at home. We expected a positive association with both the NVS and the question about books, as has been previously found, e.g., between functional health literacy and the number of books by Driessnack et al. [67]. For validation of scale E, we planned to use the MSPSS. To explore the differences between groups, we conducted the chi-square or Mann-Whitney U test (rank test) for independent groups. We used the Spearman rank-order correlation (denoted as rho) to explore the associations between variables that were not normally distributed and categorical. The GESIS Leibniz Institute for the Social Sciences was consulted twice regarding the procedure of psychometric evaluation of the 2nd draft and its results. For all descriptive and association analyses, we used STATA 15, (StataCorp LLC, Texas, TX, USA) and to perform CFA with the categorical variables, we used Mplus 8 (Muthén & Muthén, Los Angeles, CA, USA) [68].

## 3. Results

### 3.1. Stage 1 Development and Qualitative Testing

Development of the underlying conceptual framework, which was briefly described in the Methods section, started with an LR; its specification and operationalization was performed during the entire stage 1. As a second result of the LR, we identified six tools that have been applied in studies with adolescents [64,68,69,70,71,72]. Hence, we decided to use the HLS-EU-Q47-GER as a blueprint for the following reasons: the tool was available in the German language; its short version had already been tested in a quantitative study with 15-year-olds in Austria [64]; and the instrument considers health literacy not only relating to health care but also to disease prevention and health promotion [32].

We conducted CIs with nine girls and 11 boys aged 14–17 who attended various types of schools. The first round of CIs revealed limited appropriateness of the HLS-EU-Q47-GER items for adolescents. The limitations related to aspects such as unfamiliarity with some concrete and abstract terms in particular, and limited experiences regarding health care or disease prevention. Surprisingly, the respondents evaluated the health-related tasks as being “very easy” or “fairly easy”. Additionally, the findings stressed the importance of interpersonal agents, especially parents, in helping adolescents understand and judge the reliability of health information [32]. For the 1st draft of the MOHLAA tool, we adapted 16 items based on the HLS-EU-Q47-GER.

One FG was conducted with adolescents aged 14–15 (*n* = 5), and another FG was conducted with adolescents aged 16–17 (*n* = 7). Adolescents reported that the first source of health information was (in most cases) their parents and named other sources of information and support, including friends, teachers and healthcare professionals, depending on the health topic [33]. Accordingly, we added two additional scales: scale B to more accurately operationalize skills related to communication and interaction about health topics and scale E that aimed to evaluate adolescents’ perceptions of the communication skills of doctors, parents, friends and staff at school, regarding health issues. Moreover, adolescents reported that they could easily find reliable information on the internet, and they stated frequently using the internet for that purpose. However, it became evident that they did not use suitable criteria to evaluate information on the internet. This finding suggested that adolescents in our sample tend to overestimate their competencies. As this constitutes a potential source of bias when using a self-report measurement of health literacy, we also included a health knowledge scale to obtain a more robust assessment of different aspects of health literacy. While knowledge and subjective health literacy are somewhat different constructs, knowledge is regarded a constituent part of a broader understanding of health literacy, as stated by Sørensen et al. [5] and by Bröder et al. [17]. Accordingly, the questionnaire was extended with a scale including 10 knowledge questions (scale D).

Subsequently, based on the results of the LR, CIs, and FGs, we created an initial item pool that related best to our conceptual framework. The initial item pool consisted of 78 items that were derived, adapted or translated from different scales, including items from the HLS-EU-Q47-GER [34], Health Literacy Measure for Adolescents (HELMA) [70], Health Literacy Assessment Tool (HLAT-8) [41], Multidimensional Measure of Adolescents Health Literacy [71], HLS-NRW-Q [73,74], and Health Consciousness Scale [75] and one item operationalizing self-efficacy from the measure described by Schmidt et al. [25]. Health-related knowledge questions were taken or adapted among others from the health knowledge quiz described by Wallmann et al. [22], the drugcom.de quiz [76] and the food labeling quiz [77].

For the preliminary 1st draft of the MOHLAA-Q, we selected 65 items that were regarded relevant and appropriate for the age group. Based on the results of experts’ assessments and the second round of CIs, we adjusted appropriate and deleted inadequate items. For further details, see Supplementary Materials Table S1. Stage 1 was finalized with the 2nd draft including 43 items (see Table 3).

### 3.2. Stage 2: Quantitative Testing

We received data from 625 adolescents, for whom the provision of informed consent was ensured (both the participant’s and parents’ consent). The response rate for our postal survey was approximately 23% among the 2722 contacted persons. The average age of the respondents was 15.5 years (Std. = 1.12), and the distribution among the four age groups was fairly equal, as shown in Table 2.

The proportion of girls was 58.7 %. Approximately 94% of the respondents were still attending school, and 74.7% desired or had already achieved a high school certificate. The percentage of respondents with a migration background was 43.4%, which was higher than the average level for Germany (30.2–34.6% for 10–20-year-olds) [78]. All demographics characteristics were distributed statistically independently from sex (data not shown).

#### 3.2.1. Item and Reliability Analysis

The descriptive statistics of each item and the scale-level reliability coefficients as well as information on whether the items were removed from their respective scales are presented in Table 3.

##### Missing Values and Item Distribution

The frequency of missing responses ranged from 0.16% to 5.81% in scale A, from 0.48% to 0.96% in scale B, from 0.32% to 1.12% in scale C, and from 0.48% to 0.80% in scale E. Only five items had proportions of missing responses greater than 1% (items 3, 6, 15, 16 and 26); only one item had a proportion over 5%, and this item posed a somewhat a sensitive question (*How easy or difficult is it for you to understand how to use condoms*?).

The mean item scores varied depending on the scale and its response range. A mean of over 2 indicated that participants responded mostly with “easy”/“agree” or “very easy”/“strongly agree”. The mean scores on scale A varied from 1.95 to 3.53; on scale B, they varied from 2.24 to 3.04; and on scale E, they varied from 3.08 to 3.80, with a response range of 1-4. The mean scores were higher on scale C (3.38 to 4.35) due to the response range of 1–5. Most of the items had a distribution with an elongated left-hand tail (negative skew), apart from items 4, 8, 14 and 21. The gender-specific items had different difficulty levels as indicated by the mean values (mean_item15_ = 3.07 for girls vs. mean_item16_ = 3.50 for boys) and different distributions. Therefore, we excluded them from further analysis. Furthermore, the item variance varied depending on the scale, with values ranging from 0.35 (Item 5) to 1.18 (Item 25). The lowest variance was observed for items on scale A. Overall, the items were not normally distributed, and we observed ceiling effects on the item level in all four Likert-scales (see Supplementary Materials Figure S1). The response options least frequently (<10%) used by adolescents in the majority of the items were “very difficult” (scale A) and “strongly disagree” (scale B–E).

##### Discrimination and Difficulty Index

The lowest discrimination (ITC values) was observed for items on scale E (0.09 and 0.23). Some items with ITCs below 0.30 (item 24–25 and 27) were also on scale C, which indicates that these items had low correlations with the remaining items on the same scale. The items with the highest discrimination on average were on scale A. 

We computed DIs for scale D based on the mean item scores (proportion of correct answers). The average DI was 62%, and the DIs varied from 32% to 95%. Item 33, which asked about knowledge of the emergency number, and item 35, which asked about knowledge regarding the content of medicine leaflets, were answered correctly by over 95% of the respondents; therefore, we removed them from the final scale D.

##### Reliability Analysis before the CFA

The Cronbach’s α values for scales B and C were relatively low, with values barely below 0.600. Scale E had a Cronbach’s α of 0.345, which we considered too low, so we discarded the scale. Furthermore, the reliability estimate of scale D was low, as expected (KR20 = 0.263), because of the intended heterogeneity of the tested knowledge.

Due to our criteria for poorly performing items and scales, we excluded the following: items 14-16 (scale A), item 21 (scale B), items 23 and 24 (scale C), and scale E.

#### 3.2.2. Structural Validity

After confirming the assumption about a weak relationship between the items of scale D and other scales, we excluded scale D from the CFA. With the remaining 20 items, we conducted a CFA first for scales A–C and then for the overall model with the slightly revised scales. After analysis of the modification indices and factor loadings, we slightly revised scales A and C, i.e., we allowed the correlation of the residuals of items 4 and 8. Such a correlation may be an indicator of a further latent factor or a close similarity of the item wording, which was the case here. For the other scales, the residuals were not allowed to correlate.

Table 4 shows the fit indices of the slightly revised final scales and the overall model with the internal consistency coefficients of each scale. According to the conservative cut-off values for all considered fit indices, we found a good fit to the data for the scale B. In case of the scales A and C the values of CFI, TLI and RMSEA are very close to the prespecified cutoffs or above what may be indicative of an acceptable model fit. However, the found value of the WRMR for scale A does not indicate a good model fit.

**Figure 2:**
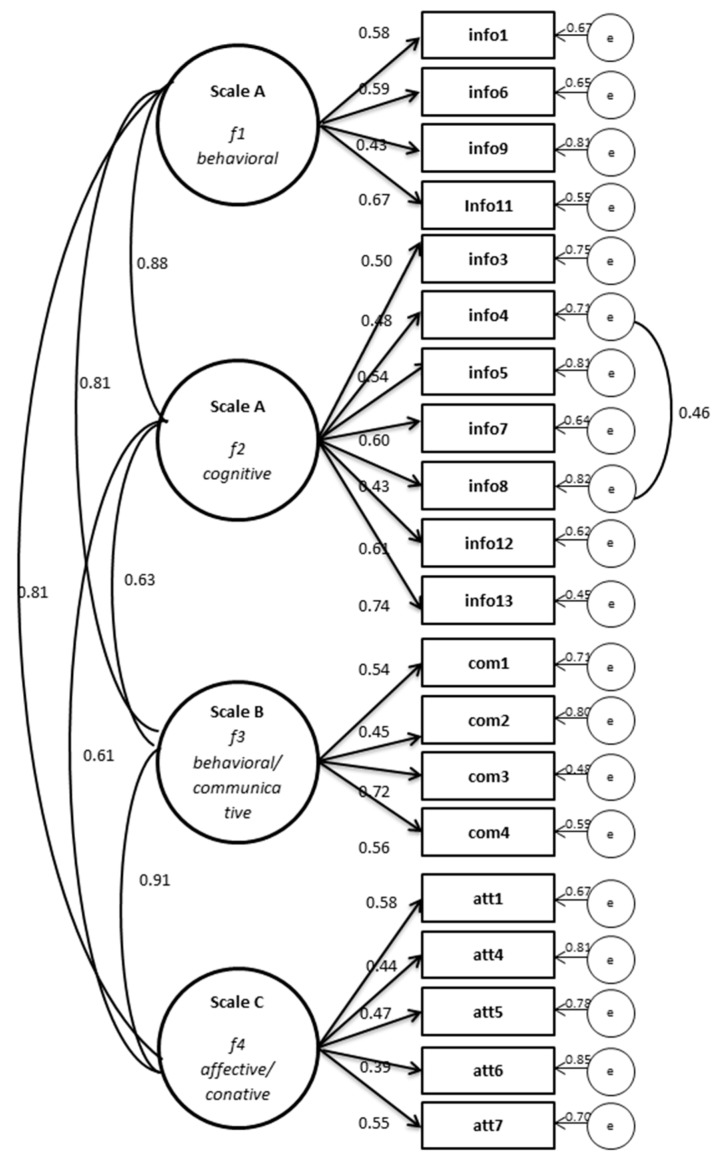
Standardized results of the CFA for the four-factor solution of the model underlying the MOHLAA-Q. Legend: Completely standardized estimates: Factor correlations, factor loadings, residual variance.

The single-factor solution for the overall model had a poor fit. In the four-factor solution, the goodness-of-fit indices showed substantial improvement. The indices were close to the values considered to indicate sufficient model fit, e.g., CFI > 0.90 and RMSEA less than or close to 0.06 [59]. Figure 2 shows the final model with a four-factor solution. The range of the standardized factor loadings (cf. Figure 2) was 0.43–0.74 for scale A, 0.45–0.72 for scale B and 0.39–0.58 for scale C. We found a high correlation coefficient for factor 3, *behavioral/communicative,* and factor 4, *affective/conative* (0.91), which indicated no clear discrimination between them.

The additional analysis for scale A with the three-factor and four-factor models proposed by Sørensen et al. [5] showed a poor fit (see Supplementary Materials Table S2), indicating that the sample data showed a better fit to our two-factor specified model (*f1 behavioral* and *f2 cognitive*) for this scale with the reduced number of items after removing poorly performing items.

#### 3.2.3. Convergent and Concurrent Validity

In Table 5, the results of the convergent validity analysis for the final scales A–C with complete cases are shown. A moderate correlation (rho = 0.459–0.528) between the MOHLAA-Q scales and the HLAT-8 indicated that our tool measures a similarly defined construct of generic health literacy. Our scales were moderately correlated (0.383–0.464) with the self-efficacy scale; the strongest effect size was found for scale A, not for scale C as we expected. A moderate association between the items measuring self-efficacy and the adapted HLS-EU-Q scale (for 4th grade pupils) was also previously found by Bollweg et al. [86]. When testing the concurrent validity, we confirmed the hypothesis regarding a negative statistical association of scales A–C with self-reported health status.

Scale D was significantly associated with the functional literacy scale NVS (rho = 0.352, *p* < 0.001), as expected, as well as with the number of books at home (rho = 0.320, *p* < 0.001). We found weak correlations between scale D and scales A–C, with values ranging from 0.099 to 0.155.

Finally, due to the results of the item and reliability analyses and the CFA, we adjusted the wording of four items that showed poor psychometric properties. We decided to revise and reinclude item 14, which we had previously excluded as a result of the item and reliability analysis. In doing so, we intended to ensure the methodological and theoretical comparability and integrity of the adapted HLS-EU-Q47-GER items with those of the original instrument for adults. The current version of the MOHLAA-Q (cf. Supplementary Materials Figure S2) consists of 29 items in four scales: scale A, “Dealing with health-related information (HLS-EU-Q12-DE-adolescents)” (12 items); scale B, “Communication and interaction skills” (4 items); scale C, “Attitudes toward one’s own health and health information” (5 items); and scale D, “Health-related knowledge” (8 items). For scales A–C, the mean raw item scores can be generated, whereby higher mean scores indicate higher self-reported skills in the corresponding dimension of generic health literacy. For scale D, assessing health-related knowledge, a sum score of the correct answers is considered. The coding of the responses can be found in Supplementary Materials Figure S2.

## 4. Discussion

We developed the first German multidimensional instrument for the assessment of self-reported generic health literacy among 14- to 17-year-olds. The MOHLAA-Q consists of four scales and 29 items, including eight questions related to health-related knowledge. For the development and validation of the MOHLAA-Q, we used a multistep study design applying several qualitative and quantitative methods. Thus, we sought to ensure a theoretical and empirical foundation of the instrument. By conducting cognitive testing of the MOHLAA-Q drafts with adolescents, we adjusted item wording for the target age group and improved the comprehensibility and acceptability of our instrument. The content validity was confirmed through the evaluation of the questionnaire drafts by the health literacy experts. Finally, the pretest results indicated the convergent validity of the individual scales. Scale A was moderately correlated with the HLAT-8 and it was found that lower health literacy mean scores are associated with poorer subjective health status; scale D was correlated, as expected, with the NVS. However, the results of the CFA (structural validity) and internal consistency analysis also identified certain limitations of the instrument and pointed to some borders in operationalization and measurement of generic health literacy. Moreover, the study revealed challenges in testing health-related knowledge and evaluating the role of social agents in processing (seeking, understanding, critical appraisal, etc.) health information.

### 4.1. Structural Validity

The MOHLAA-Q is based on a four-dimensional health literacy construct that reflects the main components of generic health literacy [17]. The goodness-of-fit indices of the overall model (scales A–C) showed better values for the four-factor solution than for the single-factor model, which may be indicative of the multidimensionality of the underlying construct. However, a closer examination of the TLI (just below the cut-off of 0.90) and high residual variance values for each item indicates that our specified age-adjusted measurement model did not show a sufficient fit to the sample data. The high correlation (0.91) between factor 3 (*behavioral/communicative*) and factor 4 (*affective/conative*) indicated no clear discrimination between the factors, suggesting that a higher-order factor may be present. From a theoretical point of view, we would not expect any common factor of those two factors (scales B and C). The localized areas of the poor fit of the specified four-factor model to the sample data must be examined in a further sample, and if necessary, the underlying conceptual framework of the MOHLAA-Q and its operationalization may require adjustment.

When comparing the MOHLAA-Q with the self-reported instruments developed for adolescents (aged 12–19) in other languages, we found instruments with similar complex structures (at least three-dimensional), e.g., the Health Literacy Measure for Adolescents (HELMA; 44 items), which is divided into eight factors (access, reading, understanding, appraisal, use, communication, self-efficacy, and numeracy) [70]; the Multidimensional Measure of Adolescents Health Literacy (22 items), which has five dimensions (interaction with the health care system, rights and responsibilities, preventive care, information seeking, and patient-provider encounter [71]; and the Health Literacy Assessment Scale for Adolescents (HAS-A; 15 items), which has three dimensions (communication about health information, confusion about health information and understanding health information) [87]. Interestingly, only one instrument, namely, the Health Literacy for School-Aged Children (HLSAC; 10 items), showed acceptable fit of a single-factor model, although the HLSAC was constructed based on five theoretical components (theoretical knowledge, practical knowledge, critical thinking, self-awareness, and citizenship) [71]. Similar to the majority of the considered instruments, the MOHLAA-Q takes into account the dimension of skills for communication and interaction with interpersonal sources, which are of great importance in this phase of life [57,79].

The intended multidimensional structure of the MOHLAA-Q has several consequences, e.g., for the length of the instrument and the interpretation of findings. Due to the multidimensionality and complexity of the generic health literacy construct, operationalization of the construct requires the consideration of multiple scales that cover single dimensions of health literacy. As a result, the instrument, i.e., the MOHLAA-Q, becomes long, which does not adhere to the pragmatic recommendations for the measurement of health literacy in this age group [57]. The multidimensionality of the MOHLAA-Q also raises the question of whether an overall index that is a sum score or a mean score for the health literacy dimensions would be an accurate indicator of generic health literacy if no single common factor is found (no unidimensionality). This fundamental point was already debated in reference to the HLS-EU-Q47-in a Norwegian validation study by Finbraten et al. [88]. Therefore, in our analysis, we considered the single scales separately by computing mean scores for scales A–C and a sum score for scale D.

Another point related to multidimensionality is that no single instrument, including the MOHLAA-Q, is capable of fully assessing all aspects of the multidimensional generic health literacy construct. However, the MOHLAA-Q addresses many health-related topics (e.g., medication adherence, nutrition, risk health behavior, physical activity) and covers many diverse health literacy aspects. The tool is intended to measure more than the perceived difficulties related to the core health literacy competencies (understanding, finding, appraising and applying health information), as in the case of the HLS-EU-Q47-GER. Rather, the MOHLAA-Q operationalizes additional core health literacy components such as health-related communication, motivation and health-related knowledge across the three health domains, as stated in the health literacy definitions proposed by Sørensen et al. [5] and Bröder et al. [17].

### 4.2. Internal Consistency

The internal consistency index (Cronbach’s α > 0.7) [89] for scale A turned out to be sufficient. However, the values for the other scales were poor, particularly for scale C that assessed affective/conative components (Cronbach’s α = 0.54). Interestingly, similar low reliability values were reported for the HELMA for a comparable scale in terms of content [70] (the self-efficacy scale with 4 items, Cronbach’s α = 0.61) and for an instrument developed by Schmidt [25] (the health-related attitudes scale, α = 0.57). This finding may be a result of the complexity of the single components (self-awareness, self-efficacy, motivation, etc.), which were represented with only five individual items. The low internal consistency could be caused by ceiling effects (meaning lower variance) on the item level which also we found for other scales. A further cause of the low value may be linked to our sample. In a very homogeneous sample in which there are hardly any differences between individuals, the reliability may be lower than in a heterogeneous sample with significant differences between persons [90]. In addition, the index of internal consistency is sensitive to the number of items (higher values with a higher number of items in a scale) and can be biased if the scale components are not essentially tau-equivalent (i.e., do not have equal factor loadings) or there is not a single common factor measured [91]. Therefore, further research is needed to examine how this scale performs in other samples and whether modified scale with additional items would result in improved measurement reliability.

### 4.3. Testing of Health-Related Knowledge (Scale D)

According to our conceptual framework, health-related knowledge is a core cognitive component of health literacy. Through the qualitative methods [32,33], we observed that adolescents tended to overestimate their skills. Thus, we integrated a few health-related knowledge questions into the instrument as a performance-based, objective measurement approach. We followed a mixed-method approach combining subjective and objective measurements, as recommended in the literature [19,92]. However, in the quantitative pretest, we found a moderate relationship of scale D with the scale measuring functional health literacy (the NVS) and only a weak relationship between scale D and A. No correlation was found with scales B and C. This finding is in line with the study by Schmidt et al. that found no statistical relationship of knowledge with the self-reported health literacy scales (measuring health-related self-efficacy, communication and attitudes) [25]. Our result may indicate that although health-related knowledge is connected to other core components of the broad concept of health literacy on the theoretical level [14,17,58], it is not necessarily closely linked to these components on the empirical level.

Testing health-related knowledge in this target group or in self-administered questionnaires in general entails some challenges and inherent limitations that also affected our study. Health-related knowledge is dynamic because of new evidence gained from health sciences, which requires ongoing updates to relevant items. A further difficulty is related to determining which health-related knowledge is essential and is practically relevant in adolescents’ lives. Hence, testing knowledge usually involves testing only some aspects of knowledge, as in the MOHLAA-Q. Another difficulty is composing questions that represent the age-relevant aspects of health-related knowledge in cognitively different age groups and that take into account gender-specific aspects of health literacy. Furthermore, with self-administered instruments that are used at home, it cannot be ensured that respondents answer the questions without any support of other persons or technical devices (which may cause measurement error and jeopardize the reliability of measurement). These constraints to testing health-related knowledge should be considered when interpreting results relating to scale D of the MOHLAA-Q. Regardless of whether health-related knowledge could be measured more comprehensively, accurately, and specifically (related to different health topics) in educational settings, a measurement of this health literacy component in a population-based survey should focus on questions relating to practical knowledge.

### 4.4. Consideration of the Role of Social Agents (Scale E)

Our results confirmed, as shown in other studies, a key role of social agents, particularly parents, in adolescents’ seeking and critical assessing health information and the complementary role of internet searches [13,79,93]. Therefore, Scale E (“Support for health-related issues by social agents”) was included in our instrument to assess this topic. However, due to the low internal consistency of this scale, the scale was discarded from the current version of the MOHLAA-Q. The low correlation of items within the scale may be a result of the variation in personal sources (doctor, parents, friends, and persons in school or work setting). Adolescents are likely to view and treat these personal sources differently based on the health subject matter [71]. Not only can adolescents’ perceptions of the health literacy of social agents vary greatly but also do these agents’ own levels of health literacy, causing indirectly low item correlation. Our findings suggest that future research should examine the nuances of accessing health information from personal sources and to what extent these sources influence adolescents’ health literacy. This suggestion is supported by other international studies that have found positive associations between parents’ educational level, literacy, and health literacy on the one hand and adolescents’ health literacy on the other hand [94,95].

### 4.5. Strengths and Limitations

In summary, our study highlights different challenges of operationalizing and measuring the broad concept of health literacy in adolescents. Referring to the latest established recommendations for the development and validation of pragmatic health literacy measures [19,57], we successfully drew on the current research in this age group and assessed the content validity of the instrument with health literacy experts. The items aim to capture characteristics of this period of life by asking about health risks and consequences of risky behavior (cf. Supplementary Materials Figure S2, item 6, 8, knowledge questions). We conducted qualitative and quantitative testing with the target group to ensure that our tool is relevant, understandable and measures what is intended to be measured (face validity). Those findings support the validity of the instrument. Further, we extensively evaluated the psychometric properties of the tool (multiple forms of validity, including structural, convergent and concurrent validity) in a large random sample. However, to ensure that our tool meets other criteria (e.g., that it is “actionable”, “broadly applicable”, “useful across settings”, etc.) [57], an ongoing development process is required. The current version of the MOHLAA-Q was applied in an additional online survey in 2019 in a representative Germany-wide sample (*n* ≈ 1200). Renewed examination of the structural validity, of internal consistency and a determination of the cut-off points and a classification scheme for the categories of self-reported health literacy assessed by the tool are planned with data on a representative sample.

The strength of measuring health literacy by self-reports is the consideration of respondents’ personal perspectives, especially, their interest and attitudes regarding seeking health-related information. However, one of the developmental characteristics of adolescents is a high drive for social recognition. Adolescents may answer questions in a socially desirable way with a tendency to overestimate their skills, which has been discussed elsewhere [32]. This possible interpretation can also be supported by ceilings effects we found for all scales in the pretest. It should be noted that ceiling effects could also be observed for similar instruments applied in adult populations [35,37,64]. Nevertheless, we believe the positive self-estimation and social desirability can partly be counteracted by ensuring that adolescents can fill out the questionnaire absolutely anonymously, outside of a context where social desirability plays a major role, e.g., in the class room. Further, the inclusion of a performance-based task (scale D) allows a performance-based estimation which is not prone to social desirability.

In our study design, we included the perspective of adolescents through qualitative testing of the drafts with *n* = 38 adolescents. Thus, we were able to adjust the item content and wording based on adolescents’ cognitive development states and needs. However, adolescents did not take part in the item selection. With only two FGs that were composed exclusively based on age-ranges, achievement of data saturation cannot be claimed. Participant inclusion in the earlier questionnaire development steps might have improved the resulting questionnaire’s acceptability and relevance for the target group [19,96]. Additionally, involvement of more experts who work with adolescents on a regular basis (e.g., teachers, nursery school teachers, school nurses) or also involvement of parents could have resulted in items that are as relevant to everyday life of adolescents as possible.

The further limitations relate to the methodology of the standardized pretest: to examine the convergent validity, we used the HLAT-8 and the NVS. The first instrument has not been validated for our target group (14 to 17-year-olds) in the German language, only in Chinese [97]. However, according to the systematic review on the quality of health literacy instruments used in children and adolescents, the HLAT-8 shows the best construct validity among the 29 considered instruments [18]. In the case of the NVS, we adapted the tool as a paper-and-pencil questionnaire. In this way, we modified the original mode of data collection, which may have altered the validity of the NVS. Only one study, from the USA, has applied the NVS as a self-administered written questionnaire among adolescents [44], and one study, from Iran, used the NVS with exclusively female adolescent students [98]. In those studies, the NVS performed well. Apart from the validation instruments, the results of the convergent validity for scale B and C should be interpreted with more caution, as only scale A met the required threshold for internal consistency. However, the values of correlation coefficients with the HLAT-8, the self-efficacy scale and the self-reported health status are similar over the scales and thus support the assumption of the convergent validity also for those scales.

Due to financial and time constraints, it was not possible to evaluate the test-retest reliability of the tool and thus measure the test consistency over time. This property is seen as an indicator of replicable and stable results [51], which will be important if the MOHLAA-Q is be applied in longitudinal studies.

Our stratified random sample included a high proportion of participants who desired or had achieved a high school certificate (approximately 75% vs. the expected 47%) [99] and a higher proportion of females than males (approximately 59% vs. the expected 50%). Such a homogeneous composition of our validation sample may have positively impacted the item difficulty indices and negatively impacted reliability values because of the smaller sample variance. Moreover, we did not collect data on non-responders, which would have provided additional insights into the validity of the results and helped to quantify sample bias.

## 5. Conclusions

Our study highlights key challenges and borders when trying to operationalize such an extensively multidimensional and broad construct as manifested by health literacy. Among those challenges was the requirement of achieving satisfactory internal consistency in all of the various scales, which was not achieved fully in this study. The most criteria of construct validity were achieved in scale A derived from the HLS-EU-items. Thus, further revision and testing in other samples is necessary to re-examine structural validity of the MOHLAA-Q and to improve the internal consistency of two scales.

The strength of our tool is that it is tailored as much as possible to the traits of health literacy in adolescence and goes beyond the assessment of perceived difficulties in dealing with health information, namely, by operationalizing health-related communication and the affective and conative components of health literacy. By complementing the tool with health-related knowledge questions, we used the recommended mixed-method measurement approach. The MOHLAA-Q reflects the multidimensionality of the health literacy construct, which is evident in the conceptualization of generic health literacy in adolescence.

## Figures and Tables

**Table 1:** 2nd draft of the MOHLAA-Q: Assessed topics and related skills.

Topic of the Scale (No. of items)	Theoretical Key Dimension	Specified, Evaluated Health Literacy Attributes
A Dealing with health-related information (16 items)	*cognitive*	understanding, appraising, critical thinking, and functional literacy skills
	*behavioral*	information seeking, application of health information
B Communication and interaction skills (5 items)	*behavioral /communicative*	skills to communicate and interact about health information
C Attitudes toward one’s own health and health information (7 items)	*affective/conative*	self-awareness, self-control, self-efficacy, motivation, interest
D Health-related knowledge (10 items)	*cognitive*	knowledge about physical activity; the health risks of alcohol use, cannabis use and smoking; the emergency number; first aid measures in case of skin burns; medication leaflets; nutrition labels; patient rights; ways of transmitting HIV/AIDS
E Support for health-related issues by social agents (5 items)	*contextual factor affecting individual health literacy*	competencies of social agents, including doctors, parents, and friends, for communication and provision of support related to health topics
43 items		

**Table 2:** Sample characteristics in stage 2 of the MOHLAA study (*n* = 625).

	Total	Percentage	Missing Values
	(*n*)	%	(*n*)%
**Demographic characteristics**			
**Sex**			(0) 0.00
Girl	(367)	58.72	
Boy	(258)	41.28	
**Age**			(0) 0.00
14 years	(159)	25.44	
15 years	(165)	26.40	
16 years	(145)	23.20	
17 years	(156)	24.96	
**Socioeconomic characteristics**			
**Are you going to school?**			(4) 0.64
Yes	(586)	93.76	
No	(35)	5.60	
**Educational level**			(9) 1.44
No high school certificate	(120)	19.20	
High school certificate	(467)	74.72	
I do not know yet	(29)	4.64	
**Family affluence**			(5) 0.80
Low	(136)	21.76	
Medium	(379)	60.64	
High	(105)	16.80	
**Migration background**			(7) 1.12
None/German	(347)	55.52	
One-sided	(109)	17.44	
Two-sided	(162)	25.92	

**Table 3:** Results of the item and internal consistency analysis analyses of the 2nd MOHLAA-Q draft (*n* = 625).

Var. Name	Scale A Dealing with Health-Related Information * *How Easy or Difficult Is It for You to?*	Source of Item	N	Missing [%] (n)	Mean	Skewness	Kurtosis	Variance	Cronbach’s α	ITC	Excluded in Step
info1	…find information about what to do when you feel ill to make yourself get better?	HLS-EU-Q47 [34] (Q1)	622	0.48 (3)	3.37	−0.50	3.11	0.36	0.775	0.46	
info2	…find information about what you can do in case of a medical emergency? *e.g., an accident, severe skin burn, alcohol poisoning*	HLS-EU-Q47 [34] (Q3)	623	0.32 (2)	3.15	−0.30	2.74	0.44	0.780	0.40	2
info3	…understand a medication leaflet	HLS-EU-Q47 [34] (Q6)	616	1.44 (9)	3.03	−0.38	2.58	0.60	0.783	0.36	
info4	…judge which information about an illness in the media you can trust and which you cannot? *media: internet, TV, radio, press*	HLS-EU-Q47 [34] (Q12)	622	0.48 (3)	2.58	0.09	2.59	0.58	0.776	0.44	
info5	...follow the instructions of your doctor or pharmacist?	HLS-EU-Q47 [34] (Q16)	622	0.48 (3)	3.48	−0.80	3.39	0.35	0.781	0.39	
info6	...find information about how you can deal with mental problems? *e.g., permanent stress, depression, being bullied, eating disorder*	HLS-EU-Q47 [34] (Q18)	618	1.12 (7)	2.84	−0.19	2.39	0.66	0.773	0.48	
info7	...understand how you can protect yourself against sexually transmitted diseases? *By sexually transmitted diseases we mean diseases such as HIV/AIDS, chlamydia infection, or herpes.*	HLS-EU-Q47 [34] (Q19)	620	0.80 (5)	3.53	−1.17	4.17	0.39	0.778	0.43	
info8	...judge whether you can trust the media when they warn you of health risks?	HLS-EU-Q47 [34] (Q28)	619	0.96 (6)	2.69	0.10	2.51	0.53	0.779	0.42	
info9	…implement advice from your family so you do not get sick?	HLS-EU-Q47 [34] (Q30)	620	0.80 (5)	3.12	−0.47	2.79	0.54	0.789	0.29	
info10	…ask your friends for health tips?	HLS-EU-Q47 [34] (Q30)	619	0.96 (6)	2.94	−0.38	2.79	0.60	0.783	0.37	2
info11	...find information about healthy behavior such as exercise and nutrition?	HLS-EU-Q47 [34] (Q32)	623	0.32 (2)	3.41	−0.63	2.40	0.42	0.774	0.47	
info12	...understand information on food packaging?	HLS-EU-Q47 [34] (Q38)	624	0.16 (1)	3.13	−0.53	2.33	0.69	0.775	0.46	
info13	...judge how what you do daily affects your health? *e.g., eating, drinking, exercise, relaxation, body care*	HLS-EU-Q47 [34] (Q43)	622	0.48 (3)	3.02	−0.28	2.21	0.64	0.770	0.52	
info14	...get involved in promoting a healthier life in your neighborhood? *e.g., more parks and sports grounds, less noise and traffic, better air quality*	HLS-EU-Q47 [34] (Q47)	620	0.80 (5)	1.95	0.59	2.90	0.66	0.794	0.23	1
	Test scale A (without item 15 and 16)								0.792 ^a^		
info15girls	...understand information about the vaccination against cervical cancer (HPV)?	HLS-EU-Q47 [34] (Q23)	367	1.09 (4)	3.07	−0.54	2.90	0.61	NC	NC	1
info16 boys	...understand how to use condoms?	HLS-EU-Q47 [34] (Q23)	258	5.81 (15)	3.50	−1.57	5.89	0.47	NC	NC	1
	**B Interactions and communication skills** ***To what extent do you agree with the following sentences?***	**Source of item**	**N**	**Missing [%] (n)**	**Mean**	**Skewness**	**Kurtosis**	**Variance**	**Cronbach’s α**	**ITC**	**Excluded in step**
com1	When you think about your last visit to the doctor, did you ask all the questions that interested you?	HELMA [70] (Q37)	621	0.64 (4)	3.01	−0.41	2.43	0.69	0.514	0.39	
com2	I chat with my friends about how one can avoid unhealthy behavior*, e.g., smoking, drinking over the limit.*	HELMA [70] (Q41)	621	0.64 (4)	2.75	−0.17	2.18	0.82	0.546	0.33	
com3	If my friends or siblings have questions about health, I can help them.	HLAT-8 [41] (Q5)	620	0.80 (5)	3.04	−0.35	3.05	0.47	0.495	0.42	
com4	It is easy for me to talk with my parents about topics about health.	Peak et al. 2011 [79]	622	0.48 (3)	3.31	−0.93	3.27	0.60	0.515	0.39	
com5	I talk to people at school or at the workplace if I have stress or problems, *e.g., with a school social worker/school teacher.*	Peak et al. 2011 [79]	619	0.96 (6)	2.24	0.33	2.00	1.03	0.604	0.22	1
	Test scale B								0.591 ^a^		
	**C Attitudes toward one’s own health and health information** ***To what extent do you agree with the following sentences?***	**Source of item**	**N**	**Missing [%] (n)**	**Mean**	**Skewness**	**Kurtosis**	**Variance**	**Cronbach’s α**	**ITC**	**Excluded in step**
att1	How much in general do you pay attention to your health?	Health Consciousness Scale [75]	621	0.64 (4)	3.38	−0.03	3.28	0.60	0.548	0.34	
att2	I am aware of my physical condition throughout the day.	Health Consciousness Scale [75]	623	0.32 (2)	4.31	−0.88	4.54	0.44	0.541	0.36	1
att3	I feel very quickly when my mood is changing.	FPSI-K [80]	623	0.32 (2)	4.35	−1.37	5.11	0.64	0.582	0.24	1
att4	I seek advice from others when I am ill.	Locus of Control about illness and health [81]	623	0.32 (2)	3.62	−0.56	2.47	1.18	0.575	0.26	
att5	It is up to me to protect myself from diseases.	Locus of Control about illness and health [81]	618	1.12 (7)	4.14	−1.05	4.52	0.67	0.549	0.34	
att6	I can influence whether or not I feel well.	Locus of Control about illness and health [81]	621	0.64 (4)	3.77	−0.64	2.84	1.06	0.575	0.26	
att7	It is important to me to know about health issues.	Health Motivation [82]	623	0.32 (2)	3.64	−0.44	2.45	1.04	0.530	0.39	
7	Test scale C								0.595 ^a^		
	**D Health-related knowledge**	**Source of item**	**N**	**Missing [%] (n)**	**Mean/item difficulty**	**Skewness**	**Kurtosis**	**Variance^a^**	**KR-20**	**ITC^a^**	**Excluded in step**
know1	How often should a young person at your age to be physically active?	Health quiz [22]	624	0.16 (1)	0.32	n.a.	n.a.	0.22	n.a.	0.05	
know2	How does it affect the body if you regularly drink a lot of alcohol?	Quiz drug com [76]	625	0.00 (0)	0.43	n.a.	n.a.	0.25	n.a.	0.09	
know3	What are the health effects for young people of consuming cannabis (marijuana, hashish) often?	Quiz drug com [76]	625	0.00 (0)	0.45	n.a.	n.a	0.25	n.a.	0.08	
know4	What is not one of the possible effects of smoking?	Quiz drug com [76]	622	0.48 (3)	0.56	n.a.	n.a.	0.25	n.a.	0.10	
know5	What phone number do you need to dial if you need an ambulance?	HLS-NRW-Q [74]	620	0.80 (5)	0.97	n.a.	n.a.	0.03	n.a.	−0.04	1
know6	How can small burns be treated?	Quiz aponet [83]	624	0.16 (1)	0.66	n.a.	n.a.	0.23	n.a.	0.13	
know7	Under which keyword does a medication package leaflet describe the undesirable effect of the medicine?	Educational material about medication leaflet [84]	624	0.16 (1)	0.95	n.a.	n.a.	0.05	n.a.	0.27	1
know8	Which ingredient is contained in the highest amount in a cocoa drink powder with the ingredients listed on the package as follows: sugar, dextrose, low-fat cocoa drink powder, emulsifying agent (lecithin), salt?	Food label quiz [77]	623	0.16 (2)	0.80	n.a.	n.a.	0.16	n.a.	0.17	
know8	We want to know if you know what your rights are. Which of the above statements is incorrect?	Massey et al. [71] (Q14)	622	0.16 (3)	0.39	n.a.	n.a.	0.24	n.a.	0.07	
know 10	How can HIV/AIDS be transmitted?	self-developed	622	0.16 (3)	0.70	n.a.	n.a.	0.21	n.a.	0.06	
	Test scale D				0.63				0.263 ^b^		
	**E Support for health-related issues by social agents**		**N**	**Missing [%] (n)**	**Mean**	**Skewness**	**Kurtosis**	**Variance**	**Cronbach’s α**	**ITC**	**Excluded in step**
cont1	Thinking about your last visit to the doctor, did your doctor explain everything to you so that you understood it?	Massey [71] (Q2)	621	0.64 (4)	3.52	−1.21	4.24	0.41	0.332	0.13	1
cont2	At school/work, there are people who help me if I have stress or problems.	self-developed	622	0.48 (3)	3.08	−0.55	2.50	0.73	0.247	0.23	1
cont4	My family helps me when I have questions about health.	HLAT-8 [41](Q6)	621	0.64 (4)	3.59	−1.55	5.40	0.39	0.245	0.23	1
cont4	If you do something that harms your health, would your friends try to dissuade you of that?	Jessor et al. [85]	621	0.64 (4)	3.31	−0.85	3.15	0.57	0.286	0.18	1
cont5	I usually can use the internet alone and undisturbed.	Peak et al. 2011 [84]	620	0.80 (5)	3.80	−2.61	9.82	0.25	0.362	0.09	1
	Test scale E								0.345 ^a^		

Legend: Source of item = from what source (instrument, quiz, educational material, survey) the item was taken with original wording or adapted or derived, and in parentheses the number of the respective item in the original instrument is shown, ^a^ = Cronbach’s α for the scale including listed items, ^b^ = KR-20 was computed for complete cases in the scale D (n = 609), ITC = item-total correlation; exclusion step 1: item and reliability analysis, exclusion step 2: confirmatory factor analyses. * The English translations of the items were done by the authors and are only illustrative.

**Table 4:** Results of the confirmatory factor analysis (*n* = 577) for scales A–C and the overall model.

MOHLAA-Q Scale	Specified Measurement Model/Factor	*No.*	*x*^2^_WLSMV_(df) *p*-value	RMSEA	CFI	TLI	WRMR	Cronbach’s α
A Dealing with health-related information	Two-factor		153.80 (42) *p* < 0.0001	0.068	0.947	0.931	1.134	0.772
	*f1 behavioral*	7						
	*f2 cognitive*	4						
B Communication and interaction skills	Single-factor *f3 behavioral/communicative*	4	2.79 (2) *p* = 0.247	0.026	0.997	0.992	0.333	0.589
C Attitudes toward one’s own health and health information	Single-factor *f4 affective/conative*	5	15.69 (5) *p* = 0.008	0.061	0.964	0.928	0.626	0.539
Overall model Scales A+B+C	Single-factor	20	868.86 (170) *p* < 0.0001	0.084	0.821	0.800	1.783	0.824
Overall model Scales A+B+C	Four-factor *f1, f2, f3, f4*	20	522.83 (163) *p* < 0.0001	0.062	0.908	0.893	1.336	0.824

Legend: df = degrees of freedom; No. = Number of items per factor; CFI = Comparative Fit Index; RMSEA = Root Mean Square Error Approximation, TLI = Tucker-Levis Index; WRMR = Weighted Root Mean Square Residual.

**Table 5:** Results of the validity (correlation) analysis for scales A–C (*n* = 577).

Scale	HLAT-8	NVS	Self-Efficacy	Subjective Heath Status
n	(565)	(555)	(573)	(576)
Cronbach’s α coefficient/KR-20	0.673	0.756 ^a^	0.856	n.a.
A Dealing with health-related information	0.528 ***	0.144 ***	0.464 ***	−0.253 ***
B Communication and interaction skills	0.484 ***	0.028 (n.s.)	0.383 ***	−0.209 ***
C Attitudes toward one’s own health and health information	0.459 ***	0.077 (n.s.)	0.383 ***	−0.207 ***

Legend: *** *p* < 0.001, n.s. = not significant, Cronbach’s α coefficient/KR-20 = were computed for the respective validation scales, ^a^ = KR-20 was computed, rho = Spearman correlation coefficient, n.a. = not available.

## Supplementary Materials

The following are available online at https://www.mdpi.com/1660-4601/17/8/2860/s1, Figure S1: Response distribution of the 2nd draft of the MOHLAA-Q items (ceiling effects), Figure S2: Final version of the Measurement Health Literacy Among Adolescents - Questionnaire (MOHLAA-Q), Table S1: Development of the MOHLAA-Q: Overview of steps, results of stage 1 (development and qualitative testing) and implications for the MOHLAA-Q drafts, Table S2: Three- and four-factor solutions for scale A according to the HLS-EU-Q model (*n* = 592).

## Abbreviations

CFA	Confirmatory Factor Analysis
CFI	Comparative Fit Index
CI	Cognitive Interview
df	Degrees of Freedom
EAs	Expert Assessments
FAS	Family Affluence Scale
FGs	Focus Groups
HLCA	Health Literacy in Childhood and Adolescence
HLS-EU-Q47-GER	German version of the European Health Literacy Survey Questionnaire, 47-item version
ITC	Corrected Item-Total Correlation
LR	Literature Review
MOHLAA-Q	Measurement of Health Literacy Among Adolescents Questionnaire
MSPSS	Multidimensional Scale of Perceived Social Support
NVS	Newest Vital Sign
HLAT-8	Health Literacy Assessment
RMSEA	Root Mean Square Error of Approximation
TLI	Tucker-Lewis Index
WHO	World Health Organization
WLSM	Robust Weighted Least Square Mean-Adjusted
WRMR	Weighted Root Mean Square Residual
