# Supplementary material for: Development and Psychometric Properties of a Questionnaire Assessing Self-Reported Generic Health Literacy in Adolescence
Source: Int J Environ Res Public Health. 2020 Apr 21;17(8):2860. doi: 10.3390/ijerph17082860 (PMC7216216; doi:10.3390/ijerph17082860)

Figure S1. Response distribution of the 2nd draft of the MOHLAA-Q items (ceiling effects).

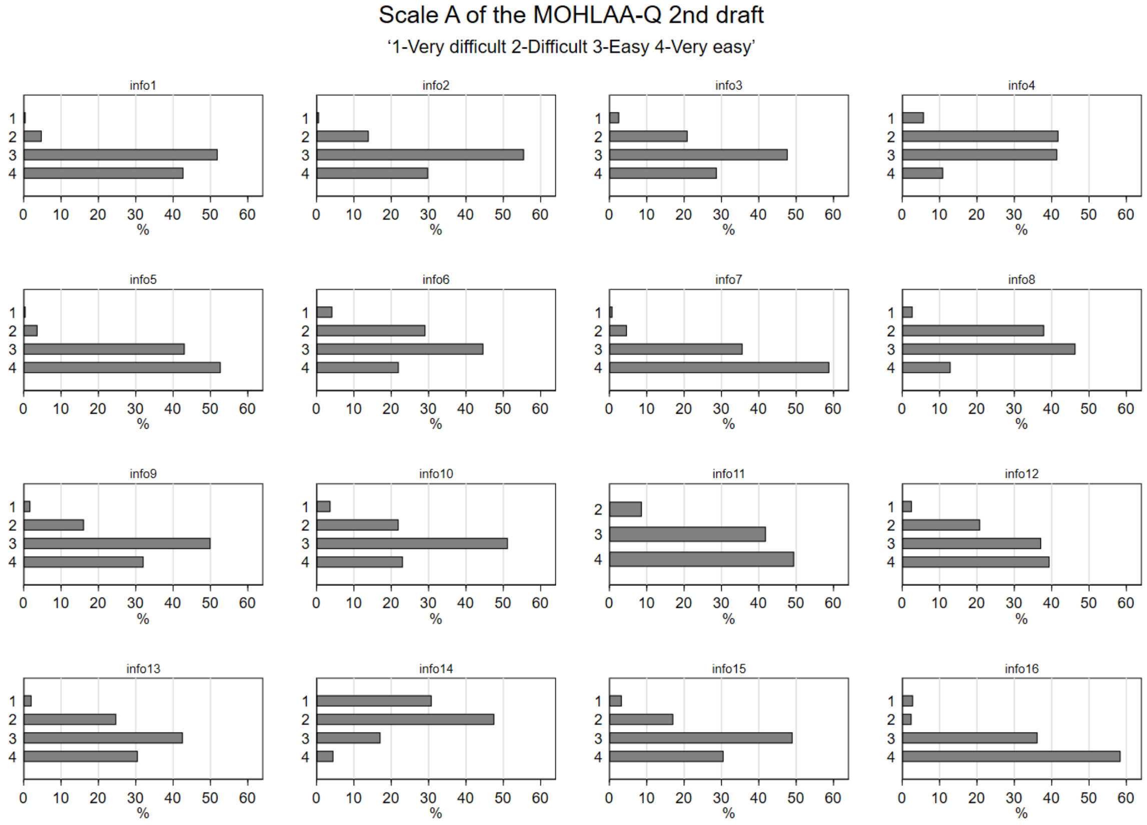

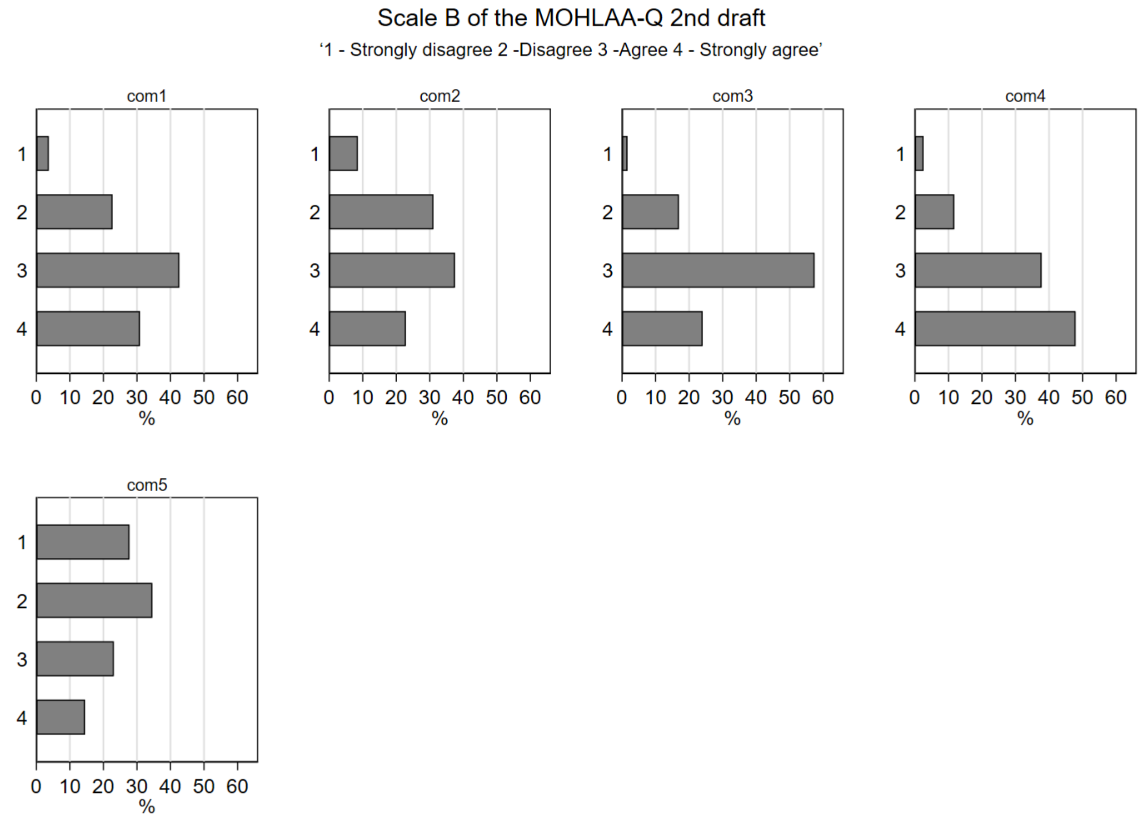

Scale C of the MOHLAA-Q 2nd draft

‘1 - Strongly disagree 2 -Agree 3 -Neutral 4 - Agree 5- Strongly agree’

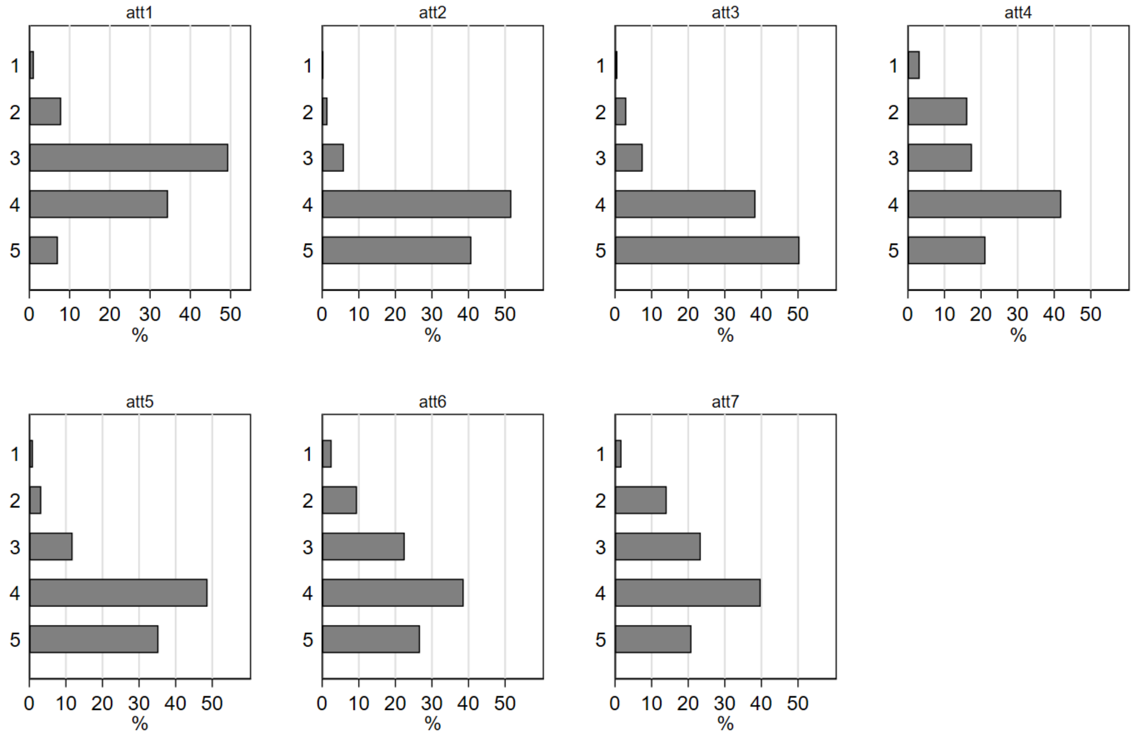

Scale E of the MOHLAA-Q 2nd draft

‘ 1 - Strongly disagree 2 -Disagree 3 -Agree 4 - Strongly agree’

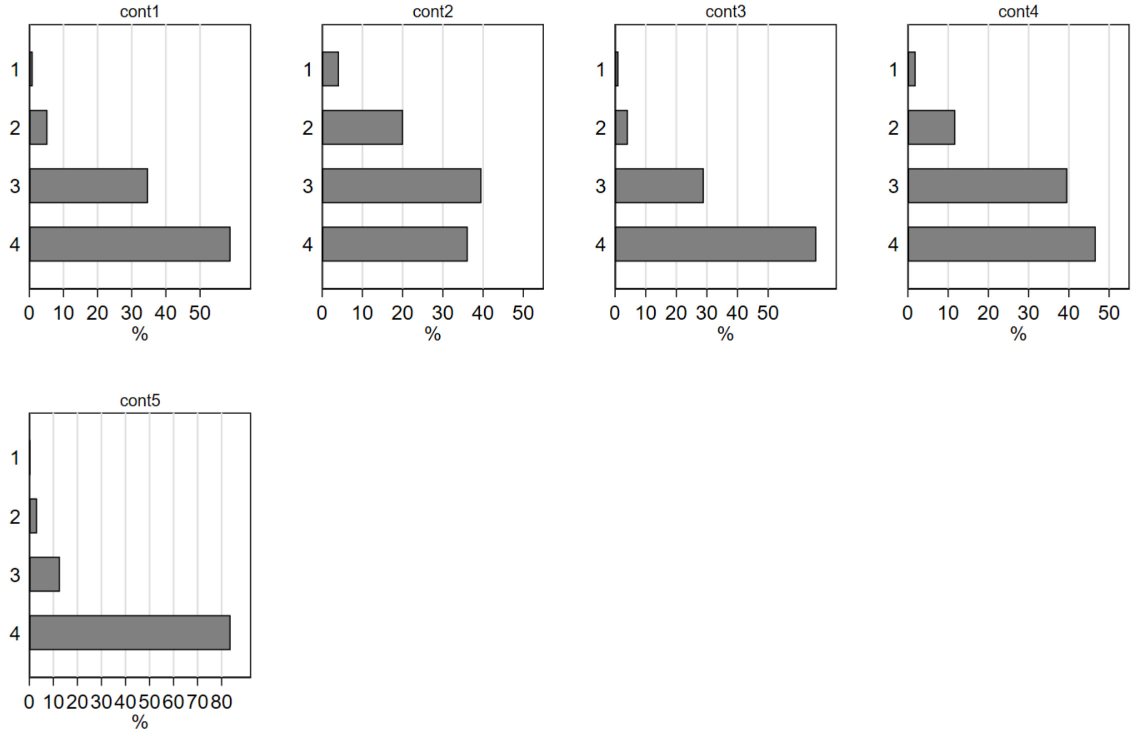

Supplement: Supplementary file 1 [file ijerph-17-02860-s001.zip › SuplementaryMaterials_S1Figure.pdf]
