# Supplementary material for: Development and Psychometric Properties of a Questionnaire Assessing Self-Reported Generic Health Literacy in Adolescence
Source: Int J Environ Res Public Health. 2020 Apr 21;17(8):2860. doi: 10.3390/ijerph17082860 (PMC7216216; doi:10.3390/ijerph17082860)
Supplement: Supplementary file 1 [file ijerph-17-02860-s001.zip › SupplemantaryMaterials_S1Table.docx]

**Table S1**. Development of the MOHLAA-Q: Overview of steps, results of stage 1 (development and qualitative testing) and implications for the MOHLAA-Q drafts.

| **Study phases/steps** | **Aims and methodological procedure** | **Main results** | **Implication for the MOHLAA-Q drafts** |
| --- | --- | --- | --- |
| Step 1  **Literature review on the definition, models and tools of health literacy in relation to adolescents** | **Aims:** To determine the conceptual framework of health literacy for the MOHLAA-Q; to generate an item pool derived from other similar health literacy measures.  **Procedure:** Search strategy: 2 databases (PubMed, Scopus) and Google scholar; in English and German | - Health literacy in childhood and adolescents is described as a dynamic, “multidimensional and complex construct” that interrelates with “social and contextual determinants” [18].   The conceptual framework of the MOHLAA-Q comprises four theoretical dimensions (factors) of generic health literacy in adolescence: cognitive, behavioral, behavioral/communicative and affective/conative competencies; consideration of a contextual factor, e.g., adolescents’ perceptions of health-related communication with physicians or family, etc. Specification of health related knowledge as a cognitive component of health literacy.   - Six tools that have been applied in studies with adolescents. - The HLS-EU-Q47-GER was determined as a blueprint for a scale operationalizing a cognitive and behavioral attributes of health literacy. | - Decision about operationalizing an age-adjusted conceptual framework of generic health literacy that includes four groups of components: cognitive, behavioral, behavioral/communicative and affective/conative. - Decision about testing the blue-print with cognitive interviews |
| Step 2  **First round of cognitive interviews** | **Aim:** To test applicability of the HLS-EU-Q47-GER for measuring the generic health literacy of adolescents.  **Procedure and data analysis:**   - Purposive sampling (n = 20) was used to achieve an equal distribution of participants regarding age groups, educational backgrounds and gender. - Standardized interview guide, verbal probing and the retrospective think-aloud technique. - 16 items were tested closely. - The interviews were audio-recorded, transcribed and analyzed using the criteria of theory-based analysis, which was derived from the model of cognitive processes. - The analysis focused on identifying terms and questions that were difficult to understand and on scrutinizing the extent to which the content of the items is appropriate for assessing adolescents’ health literacy.   The methods of the study were reported in greater detail by Domanska et al. [32] | - We interviewed 9 girls and 11 boys aged 14-17 who attended various types of schools. - The adolescent respondents were unfamiliar with some terms in the HLS-EU-Q47-GER (e.g., health risks) and provided heterogeneous interpretations of some terms (e.g., living conditions). - Respondents had limited or no experience regarding some tasks related to health care and disease prevention (e.g., the use of health screenings). - A few items seemed to be too difficult for the respondents to answer due to the high abstraction level or because they were not relevant in adolescents’ everyday life. - Despite comprehension problems with some of the HLS-EU-Q47-GER items, the respondents assessed the health-related tasks as being “very easy” or “fairly easy”. - The findings stressed the importance of interpersonal agents, especially parents, in helping adolescents understand and judge the reliability of health information.   The results of the study were reported in greater detail by Domanska et al. [32] | - 16 HLS-EU items were kept, and some of them were adjusted regarding the wording and content - Because of the observed tendency of adolescents to overestimate their health literacy skills and according to the conceptual framework, the questionnaire was extended with scale D (10 knowledge questions) as performance-based tasks. |
| Step 3  **Focus groups** | **Aim:** To explore in depth adolescents’ experiences of managing diseases, navigating health care services and promoting their own health, corresponding to the domains of HLS-EU-Q.  **Procedure und data analysis:**   - Purposive sample in Berlin (Germany) of 12 adolescents aged 14-17 chosen based on their gender, age and education level. - Based on a discussion guide with three vignettes. - Data were audio-recorded, transcribed and analyzed with the method content analysis according to Mayring. - Analysis software MAXQDA 12   The methods of the study were reported by Firnges et al. [33] | - Focus groups were conducted with adolescents aged 14-15 (n = 5) and adolescents aged 16-17 (n = 7). - Participants had limited experiences with navigating the healthcare system and managing diseases, e.g., regarding the regular intake of medication or vaccinations. - Most participants reported that their primary source of health information was their parents in case of unknown signs of illness or in case of decisions about taking medication or consulting with a doctor. - Some adolescents indicated that health-related decisions, e.g., changing to a vegetarian diet, are often made in the family context and involve parents. - Adolescents named other sources of information and support, including friends, teachers and healthcare professionals, depending on the health topic. - Adolescents stated that they could easily find reliable information on the internet, and they reported frequently using the internet for that purpose. However, it became evident based on the adolescents’ statements that they did not use suitable criteria to evaluate websites and information on the internet (tendency to overestimation)   The results of the study were reported by Firnges et al. [33] | - Addition/Development of scale B (communication/interaction) that more accurately operationalized skills related to communication and interaction about health topics - Addition/Development of scale E that aimed to evaluate adolescents’ perceptions of the communication skills of doctors, parents, friends, and staff at school regarding health issues - Confirming decision about integration of a performance-based task in measurement of generic health literacy. |
| Step 4  **Creation of the item pool/item selection** | **Aim:** An item pool that reflected the conceptual framework underlying the MOHLAA-Q.  **Procedure:** Adaptation of relevant HLS-EU-Q47-GER items for the target group, application and adaptation items from other instruments available in English or German that operationalized affective/conative and behavioral/communicative (communication and interaction skills) health literacy components and health-related knowledge; selection of items from the item pool by two project team members.  **Selection criteria:**   - Relevance of item content for the age group: Identifying the age-specific health-related topics by examining health surveys and health-related studies in Germany about disease prevalence and the prevalence of health behavior and risky behavior, the use of heath care services (e.g., health checks, vaccination) and medication use in the target group; Verifying whether the item referenced one of those identified age-specific health-related topics; - Appropriateness of the item wording (plain and concise wording): comparison of the item literacy level with that of other validated German instruments for the age group. | The item pool included 78 items.  The preliminary 1st draft of the MOHLAA-Q included 65 items.  Assignment of the items to the respective factors: scale A (f1 behavioral and f2 cognitive and), scale B (f3 behavioral/communicative) | - Verification of content validity of the operationalized conceptual framework by independent researchers - Verification of the fit of the chosen items to the framework by independent researchers - Review of the item language by independent researchers |
| Step 5  **Frist round of expert assessments** | **Aim:** To ensure the content validity of the first draft of the MOHLAA-Q.  **Procedure:** A qualitative assessment of the item language and relevance for operationalizing adolescents’ health literacy by 3 researchers with backgrounds in education/teachers (2 experts) and medicine (1 expert).  If an item was rated as “important” and “linguistically appropriate”, it was retained in the final 1st MOHLAA-Q draft. | - The preliminary 1st draft was rated in general as a “very good” instrument to assess the comprehensive construct of health literacy. - The majority of the items were rated as having a “good fit” or being highly relevant. - The experts recommended phrasing items about sexual health by referring to adolescents’ needs in their everyday lives, e.g., contraception and protection against sexually transmittable disease. | - 11 items were discarded and 54 items were retained in the final 1st draft of the MOHLAA-Q. - The wording of 42 items was adjusted. |
| Step 6  **Second round of cognitive interviews** | **Aim:** To identify understanding problems and sources of response errors in the 1st draft of the MOHLAA-Q;  **Procedure and data analysis**   - Sample: n = 18 cognitive interviews in Berlin, Germany; random sample of addresses from the resident register in Berlin (n = 310); participants were contacted via a mailed invitation. Additional recruitment of study participants from youth clubs, girls’ clubs, and sports clubs in socially disadvantaged city districts, to skew our sample toward participants with lower educational backgrounds. - Standardized interview guide; targeted testing of 23 of the 54 items from the 1st draft of the MOHLAA-Q using verbal probing and the retrospective think-aloud techniques; the interviews were audio-recorded, transcribed and analyzed using the criteria of theory-based analysis, which was derived from the model of cognitive processes. | - Eleven boys and 7 girls aged 14-17 years participated. Nine of the 18 interviewees had achieved or aimed for low- or medium-level secondary school certificates, and the other 9 interviewees had high school certificates. - The majority of the tested items and answer options were well understood by the participants. - Identification of some difficult or ambiguous terms and expressions (e.g., health problems, neighborhood, HPV vaccination, medical assistants, to be physically active). For these terms, we paraphrased, gave examples or used colloquial terms. - Cognitive interviews indicated which questions should be gender-specific.   Identify the poorly performing knowledge questions that either could not be answered by all four age groups or by both genders (high percentages of “do not know” responses), were evaluated as “very easy” or “very difficult” by the vast majority of adolescents.   - Participants reported the most difficulty understanding the adapted HLS-EU-Q47-GER items and noted that the item content in this scale seemed to be repetitive. They referred to their lack of experience with health care-related topics, which resulted in them providing answers based more on their knowledge and less on their own experience. - The adolescents found the questionnaire to be varied and considered it "feasible" to complete. | - Adjusting the response format of the knowledge questions to a single-choice question format. - Wording of one separated question regarding sexuality for girls and one question for boys - Adjusting the items’ wording and content and reducing the number of items to 43. |
| Step 7  **Second round of expert assessments** | **Aim:** To ensure the content validity of the preliminary 2nd draft of the MOHLAA-Q.  **Procedure:** The experts scored the clarity of the wording of each item and the item relevance regarding the generic health literacy construct on a 4-point scale (1-very clear/very important, 2-clear/important, 3-less clear/less important, 4-not clear/not important). The same scoring scheme was used to assess the clarity and relevance of the individual scales of the 2nd draft of the MOHLAA-Q.  The educational backgrounds of the experts included education/teacher (2 experts), medicine (2 experts), public health and health science (1 expert). | - Four of the five experts rated the dimensions of the preliminary 2nd draft of the MOHLAA-Q and the single items as "very important" or "important" for the measurement of generic health literacy in adolescence. - One expert argued that health-related knowledge should be reflected in a separate scale from the generic health literacy construct. The expert pointed out that knowledge can be measured more validly in school settings than in self-administered surveys. | - Keeping scale D - Improvement of the item wording. - No item was removed from the 2nd draft that included 43 items. |
