# Supplementary material for: Development and Psychometric Properties of a Questionnaire Assessing Self-Reported Generic Health Literacy in Adolescence
Source: Int J Environ Res Public Health. 2020 Apr 21;17(8):2860. doi: 10.3390/ijerph17082860 (PMC7216216; doi:10.3390/ijerph17082860)
Supplement: Supplementary file 1 [file ijerph-17-02860-s001.zip › SupplemantaryMaterials_S2Table.docx]

**Table S2.** Three- and four-factor solutions for scale A according to the HLS-EU-Q model (n = 592).

| **Specified measurement model** | **No.** | **X^2^WLSMV (df)  p-value** | **RMSEA** | **CFI** | **TLI** | **WRMR** | **Problem** |
| --- | --- | --- | --- | --- | --- | --- | --- |
| f1 health care | 5 | 454.27 (74)  p<0.0001 | 0.031 | 0.868 | 0.838 | 1.680 | The latent variable covariance matrix (psi) is not positive definite.  Correlation greater 1 between f1 and f2 (1.093) |
| f2 disease prevention | 4 |  |  |  |  |  |  |
| f3 health promotion | 5 |  |  |  |  |  |  |
| f1 find | 5 | 450.78 (71) p<0.0001 | 0.095 | 0.868 | 0.831 | 1.648 |  |
| f2 understand | 3 |  |  |  |  |  |  |
| f3 access | 3 |  |  |  |  |  |  |
| f4 apply | 3 |  |  |  |  |  |  |

n - Complete cases for scale A; items 1-14 were included in the CFA analysis. Legend: df = degrees of freedom; No. = Number of items per factor; CFI = Comparative Fit Index; RMSEA = Root Mean Square Error Approximation, TLI = Tucker-Levis Index; WRMR = Weighted-Root-Mean-Square Residual
